# Effects of single and multiple sessions of lower body diastole-synchronized compressions using a pulsating pneumatic suit on endothelium function and metabolic parameters in patients with type 2 diabetes: two controlled cross-over studies

**DOI:** 10.1186/s12933-022-01710-6

**Published:** 2022-12-22

**Authors:** Paul Valensi, Nicolas Barber-Chamoux, Amel Rezki, Céline Lambert, Bruno Pereira, Christian Dualé, Dominique Delmas, Martine Duclos

**Affiliations:** 1 Present Address: Endocrinology, Diabetology and Nutrition Unit, AP-HP, Jean Verdier Hospital, Sorbonne Paris Nord University, CRNH-IdF, CINFO, Bondy, France; 2 Department of Cardiology, CHU Clermont-Ferrand, Clermont-Ferrand, France; 3 Biostatistics Unit, DRCI, CHU Clermont-Ferrand, Clermont-Ferrand, France; 4 Clinical Investigation Center (INSERM CIC1405), CHU Clermont-Ferrand, Clermont-Ferrand, France; 5 SESAME SAS, Charenton-Le-Pont, France; 6 Department of Sports Medicine and Functional Explorations, CHU Clermont-Ferrand, INRAE, UNH, CRNH Auvergne, Clermont Auvergne University, Clermont-Ferrand, France

**Keywords:** Type 2 diabetes, Endothelial function, Shear stress, Pulsating compressions, Metabolic effects

## Abstract

**Background:**

Endothelium function is often impaired in patients with type 2 diabetes. We hypothesized that by improving endothelial function using diastole-synchronized compressions/decompressions (DSCD) to the lower body may improve the metabolic profile. The objective of this research was to evaluate the effects of single and multiple DSCD sessions on microcirculation, endothelium function and metabolic parameters of patients with type 2 diabetes.

**Methods:**

Two monocentric, controlled, randomized cross-over studies (Study 1 and Study 2) were performed. In Study 1, 16 patients received one 20 min DSCD and one simulated (control) session at 2 week intervals; continuous glucose monitoring and cutaneous blood flow were recorded continuously before, during and after DSCD or Control session; other vascular assessments were performed before and after DSCD and control sessions. In Study 2, 38 patients received 60 min DSCD sessions three times/week for three months followed by a 4–6 week washout and 3 month control period (without simulated sessions); vascular, metabolic, body composition, physical activity and quality of life assessments were performed before and after 3 months.

**Results:**

Both studies showed significant, multiplex effects of DSCD sessions. In Study 1, cutaneous blood flow and endothelium function increased, and plasma and interstitial glucose levels after a standard breakfast decreased after DSCD sessions. In Study 2, cutaneous endothelium function improved, LDL-cholesterol and non-HDL cholesterol decreased, extra-cell water decreased and SF-36 Vitality score increased after 3 months of DSCD sessions.

**Conclusions:**

Our findings support the beneficial effect of DSCD on the endothelium and show concomitant beneficial metabolic and vitality effects. Future clinical trials need to test whether DSCD use translates into a preventive measure against microvascular diabetic complications and its progression.

*Trial registration* ClinicalTrials.gov identifiers: NCT02293135 and NCT02359461.

## Background

Endothelial function is often impaired early on patients with type 2 diabetes and plays a pathogenic role in the disease [1, 2]. Endothelial cells (ECs) undergo local mechanical strains generated by an increase in blood velocity and longitudinal shear forces, known as shear stress. Through mechanoreceptors, ECs sense small variations in blood-induced shear stress which translate a physical force into biochemical signaling events, leading to changes in gene expression and increase vasoactive-amines, nitric oxide (NO) bioavailability and EC remodeling [3–8].

Enhanced external counter pulsation (EECP) is a non-invasive method that repeatedly applies high pressure (300 mmHg) compressions on the lower body and has primarily shown to increase coronary blood flow [9] and flow-mediated dilation (FMD) in patients with chronic angina [10]. In type 2 diabetes, it has shown to improve insulin sensitivity and decrease plasma glucose in patients [11]. However, this method has not been well tolerated and has induced adverse events such as muscle, leg and back discomfort/pain and skin abrasion [12]. As a result, a pulsating pneumatic suit applying diastole-synchronized compression/decompression (DSCD) cycles at pressure levels lower than those of EECP was developed. Its purpose has been to non-invasively stimulate the endothelial layer of the lower body via a purely mechanical effect.

Cutaneous blood flow (CBF) measurement is an indicator of the microcirculatory function of the skin as well as other vascular beds [13–15]. One study evidenced a two-fold increase of CBF on the forearm and was measured at away from where a DSCD pulsating suit was worn by healthy volunteers for a duration of 20 min [16]. To date, however, the metabolic effects of this treatment are unknown. We hypothesized that by improving endothelial function using DSCD to the lower body may improve the metabolic profile. The objective of this study was to evaluate the effects of single and multiple DSCD sessions on microcirculation, endothelium function and metabolic parameters on type 2 diabetes patients via two independent studies (Study 1 and Study 2).

## Methods

### Study population

Patients with type 2 diabetes were recruited from the University Hospitals in Bondy (Study 1) and Clermont-Ferrand (Study 2) in France. Type 2 diabetes was asserted on the family history of diabetes, relative insulin deficiency, insulin resistance, and the absence of biological evidence for autoimmune destruction of beta cells and other causes of diabetes. The inclusion criteria were patients: 18–75 years old, with a body mass index (BMI) equal to 20–45 kg/m^2^ and HbA1c levels between 6 to 11%. The patients had to be treated with oral glucose lowering drugs, insulin and/or incretin and without any known cardiovascular history. Exclusion criteria were venous thrombosis, peripheral artery occlusive disease, peripheral neuropathy, uncontrolled hypertension, kidney, heart and/or respiratory failure.

### DSCD sessions

The DSCD sessions were performed with a portable Stendo3^®^ device (Stendo Company, Louviers, France) which used an inflatable suit positioned on the lower limbs and abdomen and generates low-level compressions synchronized with diastole followed by decompression during systole as detected via SpO_2_ finger pulse oximeter signals. The device was composed of an electro-pneumatic console that controls the inflatable clothing. It is composed of six independent multilayer cells (including a gelatinous layer) applied to each leg and has individual pneumatic controls. During each session, the operator placed the finger oximeter on the patient who was in either a prone or semi-seated position then closed the suit on the subject’s lower body using self-adhesive straps in order to tailor it to the body without being too tight.

### Study design and measures

**Fig. 1:**
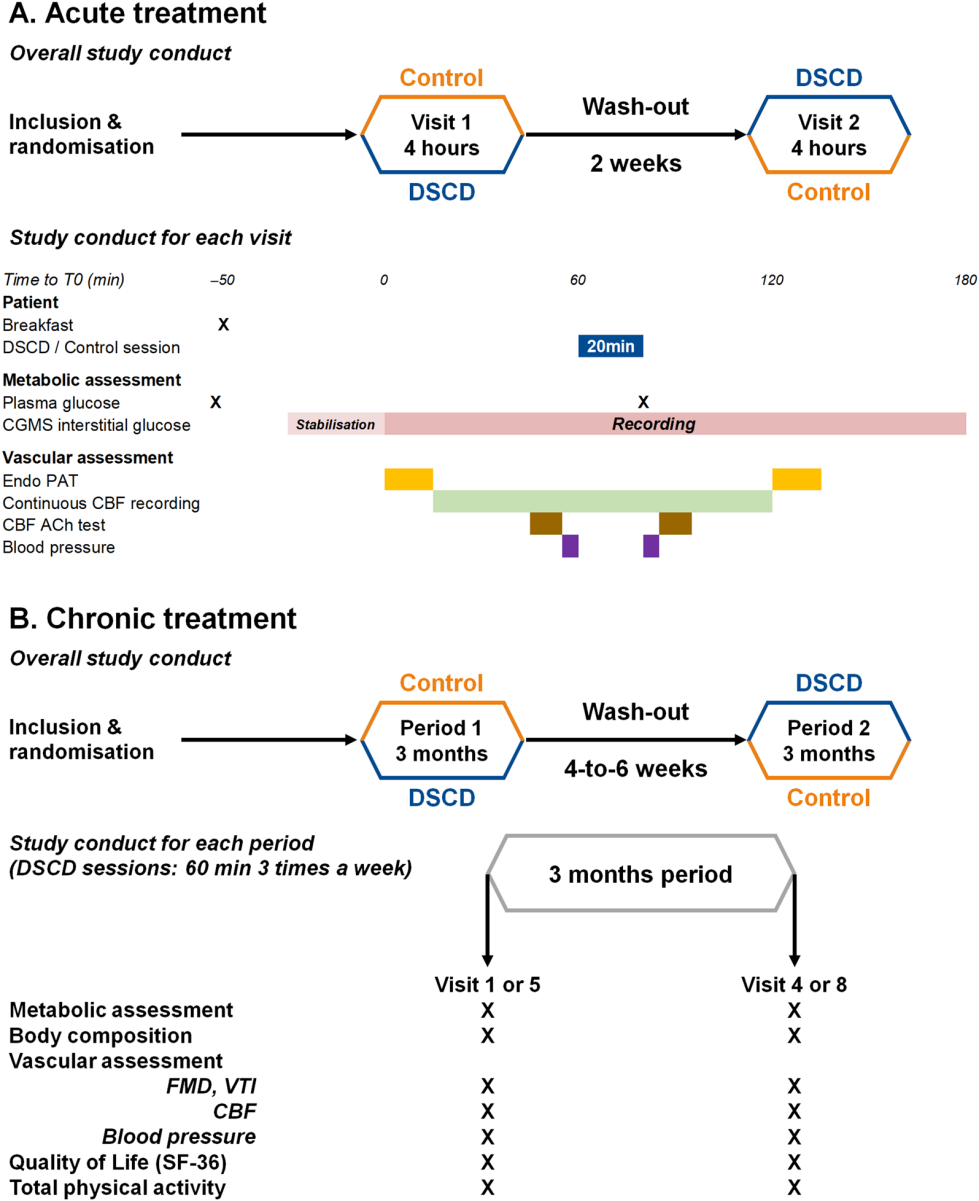
Study design and measures for both Study 1 and 2. **A**: Study 1 with 16 patients taking a single 20 min DSCD/control session. ACh; acetylcholine, control; simulated session (diastole synchronized compression/decompression session at 5 mmHg), CBF; cutaneous blood flow, DSCD; diastole synchronized compression/decompression session at 65 mmHg, RHI; reactive hyperaemia index. Arm-1: one DSCD visit followed by one control visit. Arm-2: one control visit followed by one DSCD visit. RHI was measured with Endo-PAT^®^, CBF was measured with Laser Doppler flowmetry (LDF), blood pressure was measured with SphygmoCor^®^. Blood samples were taken at fasting and again after the end of the DSCD/control session. Breakfast was eaten 50 min prior to starting CGMS stabilized recording (T0) (indicated in dark pink). Light pink corresponds to the 45 min needed to achieve CGMS stabilization. DSCD/control sessions started one hour (T60) after the start of CGMS recording and lasted for 20 min. Vascular assessment was performed twice: before and after DSCD/Control session. **B**: Study 2 with 38 patients taking three 60 min DSCD sessions in a week for three months*.* CBF; cutaneous blood flow, control; no session was performed, DSCD; diastole synchronized compression/decompression session at 65 mmHg, FMD; flow-mediated dilation, SF-36; 36-Item Short Form Survey (a self-questionnaire to measure health status), VTI; speed of hyperaemia response. Arm-1: three 60 min DSCD sessions a week within three months followed by a 3-month control period (reverse for Arm-2). Blood pressure was measured with SphygmoCor^®^. CBF was measured with Laser Doppler flowmetry (LDF)

Both studies were single-center, controlled and randomized with two cross-over arms (Arm-1 and Arm-2). In Study 1, Arm-1 cohorts received one 20 min DSCD session followed by a simulated (control) DSCD session at 5 mmHg 2 weeks later (Fig. 1A). A reversed sequence was carried out for patients in Arm-2. Plasma glucose was measured during fasting and again immediately after the DSCD control sessions. A standard breakfast including unsweetened coffee or tea, orange juice and white bread with jam (75 g of carbohydrates, 5.4 g of proteins, 0.9 g of lipids and 1.9 g of fibers) [17] was consumed 50 min before starting continuous glucose monitoring (CGM) and recorded (T0) over the following three-hour period. CBF measurement started 15 min after T0 and was performed continuously for 105 min. Other vascular assessments were performed before and after both DSCD and control sessions according to a mirror schedule centered on a session.

In Study 2 (see Fig. 1B), Arm-1 patients underwent three 60 min DSCD sessions per week for 3 months (Visits 1–4) followed by a 4-to-6 week washout and a subsequent 3 month control period (Visits 5–8) but without simulated sessions. The reverse sequence was carried out for patients in Arm-2. Vascular, metabolic, body composition, physical activity and quality of life assessments were performed before and after three months (24 h after the last DSCD session for the period with DSCD).

In Study 1, an assessment of microcirculation was completed by measuring CBF, endothelium function by CBF response to acetylcholine (ACh) by iontophoretic administration on the forearm, and reactive hyperemic index (RHI) in addition to assessing other biological parameters including plasma and interstitial glucose. Study 2 assessments included post-occlusive reactive hyperemia (PO-RH) CBF on the palmar hand, PO-RH brachial FMD and speed of hyperemia response (VTI), plasma glucose, insulin, lipids, and HbA1c and body composition measurements. An SF-36 Quality of Life questionnaire [18] was completed and an assessment of physical activity based on the International Physical Activity Questionnaire (IPAQ) [19].

### Analytical methods

#### Vascular measurements

In Study 1, microvascular blood flow response and microvessel/small artery endothelial function were assessed in response to one DSCD session.

- The forearm cutaneous blood flow response was assessed by measuring the changes in capillary CBF using laser-Doppler flowmetry (Periflow 5000^®^, Software Version 2.55, Perimed, Stockholm, Sweden) and A 481–1 sensor placed on the forearm. The CBF area under the curve (AUC) was calculated during the 20 min session (AUC-20 min) continuing until 20 and 30 min after the end of session (AUC-40 min and AUC-50 min).
- Changes in the endothelial function were evaluated by measuring: (i) the forearm microvascular response to ACh iontophoresis. CBF changes using the same equipment/sensor/location were measured after cutaneous administration of 0.2 ml of 2% ACh (Miochole^®^, Novartis) achieved through 0.2mAmp 25 s iontophoretic pulses [20–22] and by calculating the 5 min incremental CBF AUC after ACh administration (AUC5min) and (ii) the finger small arteries response to occlusion reperfusion. The reactive hyperemia was measured by recording finger arterial pulsatile volume changes using plethysmographic biosensors (Endopat2000^®^ system, Itamar Medical, Caesarea, Israel) after occluding brachial artery blood flow for five minutes. RHI represents the post-to-pre occlusion signal ratio of the occluded side normalized to the control side.

In Study 2, macro and microvessel endothelial function were assessed in response to 3 month repeated DSCD sessions.

- The brachial artery dilation and speed of hyperaemia in response to occlusion reperfusion was assessed by measuring the FMD (performed as previously described [23]) on the left brachial artery on the longitudinal plane above the antecubital fossa using a 7–12 MHz high-resolution ultrasound system (Vivid S5^®^, GE Healthcare, Velizy-Villacoublay, France). A mechanical arm device (Vascular Imaging^®^, Leiden, Netherlands) was used to obtain stereotactic positioning of the transducer. Images that were recorded at baseline for 10 s and again 2 minutes after the release of forearm ischemia (220 mmHg for 5 min) were digitized for a subsequent blinded analysis using automated edge detection software (Hemodyn 3 M^®^, Dinap SRL, Buenos Aires, Argentina). The FMD result was expressed as the percentage increase in the diameter of the brachial artery from baseline to maximal dilatation occurring 30–90 s after cuff release. The speed of hyperemia response was measured during the 10 s post-occlusion period and was obtained by the time/speed integration of the spectrum of the first complete flow after the occlusion release (velocity time integral (VTI). Brachial SSRH (Shear Stress in response to Reactive Hyperaemia) was calculated by multiplying the viscosity (8 ×0.035) and the speed normalized by the heart rate, divided by the diameter [24].
- The microvessel endothelial function was assessed by the palmar hand microvascular response to occlusion reperfusion; PO-RH CBF AUC for 5 minutes (AUC5min) was recorded after a 5 min brachial artery occlusion using Periflow 5000^®^ and a 457/PI sensor placed on the palmar hand.

Arterial blood pressure was measured using SphygmoCor CPV^®^ for Study-1 and XCEL^®^ (Atcor Medical, Sydney, Australia) for Study 2. Radial tonometry calibrated to brachial cuff pressures was used to estimate central aortic pressure with the SphygmoCor built-in transfer function [25]. Only measurements having an operator index above 80 were used.

#### Metabolic measurements

In Study 1, in addition to plasma glucose (both during fasting and at the end of the control or DSCD session), interstitial glucose (i-glucose) was measured using a CGM device blinded to the participant (Navigator II^®^, Abbott Laboratories, Issy-les-Moulineaux, France) placed on the patient’s arm. i-glucose decremental AUCs starting from T60 (start of session, which represented a period of time 110 min after breakfast) for 1 hour (T60-120) and 2 hours (T60-180) were calculated. In Study 2, venous blood samples were obtained under fasting conditions. Plasma total cholesterol, HDL-cholesterol, triglycerides, insulin and glucose were measured using standard laboratory procedures. LDL-cholesterol (Friedwald formula), non-HDL cholesterol and triglycerides-glucose (TyG = Ln [Triglycerides (mg/dL) ×Glucose (mg/dL)/2]) index were calculated. Body composition (fat mass, lean mass, extra and intra-cell water) was evaluated using a bioelectrical impedance device (Bodystat^®^ Quadscan, EuroMedix, Köln Germany).

### Statistical analyses

Statistical analyses were performed using SPSS V20 (IBM SPSS Statistics, Chicago, IL, USA) and Stata software V15 (StataCorp, College Station, TX, USA). Categorical data were expressed as frequencies and percentages and continuous data as mean ± SD or median [1st quartile; 3rd quartile] according to the statistical distribution.

Baseline comparisons of the two randomized groups (DSCD/Control vs Control/DSCD) were performed using the chi-squared test or Fisher’s exact test for categorical variables and the Student’s *t* test or Mann–Whitney test for quantitative variables. Due to the cross-over design, linear mixed-effects models were performed with (i) patient as random-effect (to consider between- and within-subject variability) and (ii) with the following independent variables: period and sequence of the cross-over, group (control or DSCD), time (before and after sessions), and interaction “group x time” (p-value expressed by p^i^). Subgroup analyses (Control or DSCD) were similarly carried out but with the following independent variables: period and sequence of the cross-over and time (before and after sessions) (p). The normality assumption of residuals from these estimated models was analyzed with the Shapiro–Wilk test. If appropriate, a logarithmic transformation of the dependent variable was performed to achieve normality. Finally, Spearman’s correlation coefficients (denoted *r*) were calculated between the i-glucose decremental AUC T60-180 and ACh CBF AUC-5 min (before and after either Control or DSCD sessions). The tests were two-sided with a type I error set at 5%. Since analyses of secondary endpoints were exploratory, the individual p-values were reported without systematically applying mathematical correction.

Based on a 97% increase in resting CBF (p < 0.01) reported after a 20 min DSCD session [16] and assuming that such difference was expected in patients with type 2 diabetes between the DSCD and control groups, 16 patients were considered relevant in detecting a significant between-group CBF difference in Study 1 (two-sided type I error of 5%, 80% power).

In Study 2, FMD was the primary endpoint. According to previous results [26], a 5% (± 2.7%) mean ± SD FMD defined the sample size. Thus, forty-four patients would allow the detection of an absolute 2% FMD difference between groups (two-sided type I error of 5%, power of 90%, intra-individual correlation coefficient of 0.5).

## Results

### Patient characteristics

A total of 16 and 38 patients with type 2 diabetes aged 53.3 ± 11.4 to 62.4 ± 7.6 years with HbA1c 7.1 ± 0.8 and 7.2 ± 1.2% were included in Study 1 and Study 2 respectively. The sex of the patients was 6:10 and 21:17 male to female ratio respectively and the mean BMI was 27.8 ± 3.5 in Study 1 and 32.8 ± 5.7 kg/m^2^ in Study 2.

Patients were treated by at least one glucose lowering drug (metformin, gliptin and/or sulphonylureas). Three among the 16 in Study 1 and 14 among the 38 patients in Study 2 had insulin treatment. Study 1 patients took less antihypertensive drugs than those in Study 2 (mean 0.7 drug vs 1.63) and were less frequently taking statins at inclusion (37.5% vs 60.5%). Blood pressure and overall biological parameters were comparable in the two studies. No statistical differences for the baseline parameters were found between the arms in either of the two studies except for triglyceride levels in the Study 2 cohorts (Tables 1, 2). Treatments remained unchanged during the studies with the exception of four patients for whom diabetes treatments were modified and for two patients who started statins during Study 2. In these patients, metformin was added at the end of the control sessions and stopped before the beginning of the DSCD sessions. Therefore, it is unlikely that these treatment changes could have modified the positive effects of the DSCD sessions.

**Table 1:** Patient characteristics for study 1

	Total	DSCD/Control	Control/DSCD	p-value
	(n = 16)	Arm 1 (n = 8)	Arm 2 (n = 8)	
Gender (Male/Female)	6/10	4/4	2/6	
Age (years)	53.3 ± 11.4	52.1 ± 14.9	54.5 ± 7.3	0.705
Weight (kg)	78.5 ± 15.1	76.9 ± 12.1	78.7 ± 13.2	0.860
Height (cm)	166.3 ± 8.6	164.9 ± 6.7	165.8 ± 7.5	0.958
Body mass index (kg/m^2^)	27.8 ± 3.5	28.0 ± 4.9	27.9 ± 4.1	0.909
Waist circumference (cm)	93.4 ± 9.3	90.3 ± 14.3	91.8 ± 11.8	0.612
Smoker status	0 (0.0)	0 (0.0)	0 (0.0)	NA
HbA1c (%)	7.07 ± 0.77	6.85 ± 0.78	7.29 ± 0.74	0.272
HbA1c (mmol/mol)	53.78 ± 8.42	51.37 ± 8.53	56.18 ± 8.01	0.272
Diabetes duration (years)	9.0 [5.0; 13.0]	9.0 [3.5; 11.5]	8.0 [7.0; 15.0]	0.697
Fasting plasma glucose (mmol/L)	8.30 ± 2.09	7.92 ± 2.31	8.69 ± 1.87	0.532
Total cholesterol (mmol/L)	4.45 ± 1.07	4.15 ± 1.03	4.85 ± 1.08	0.248
HDL-cholesterol (mmol/L)	1.20 ± 0.59	1.02 ± 0.39	1.45 ± 0.75	0.194
LDL-cholesterol (mmol/L)	2.47 ± 0.93	2.24 ± 0.65	2.78 ± 1.21	0.303
Triglycerides (mmol/L)	1.31 ± 0.77	1.52 ± 0.70	1.04 ± 0.83	0.268
Non-HDL cholesterol (mmol/L)	3.24 ± 1.29	3.13 ± 1.06	3.40 ± 1.64	0.714
Plasma creatinine (µmol/L)	69.0 ± 14.4	72.4 ± 13.8	65.1 ± 15.1	0.349
Glomerular filtration rate (CKD, mL/min/1.73 m^2^)	98.3 ± 19.4	97.3 ± 24.0	99.6 ± 14.3	0.961
Aortic SBP (mmHg)^a^	120.6 ± 15.6	116.1 ± 11.1	124.9 ± 19.0	0.578
Aortic DBP (mmHg)^a^	83.9 ± 11.6	81.3 ± 4.2	86.6 ± 15.6	0.644
Aortic pulse pressure (mmHg)^a^	37.6 ± 10.1	34.9 ± 9.1	40.3 ± 10.8	0.570
Rest CBF (PU)	4.97 [2.67; 7.41]	4.84 [3.23; 6.43]	5.11 [2.67; 7.41]	0.740
Insulin treatment	3 (18.8)	1 (12.5)	2 (25.0)	0.554
Statins	6 (37.5)	3 (37.5)	3 (37.5)	1.000
Fibrates	1 (6.3)	1 (12.5)	0 (0.0)	0.334
Antiplatelet treatment	5 (31.3)	3 (37.5)	2 (25.0)	0.619
ACE-inhibitor	2 (12.5)	1 (12.5)	1 (12.5)	1.000
ARB	2 (12.5)	2 (25.0)	0 (0.0)	0.149
Beta-blockers	2 (12.5)	1 (12.5)	1 (12.5)	1.000
Calcium-channel blockers	3 (18.8)	1 (12.5)	2 (25.0)	0.554
Other antihypertensive drugs	2 (12.5)	1 (12.5)	1 (12.5)	1.000

Data are presented as numbers, percentages (%), mean ± standard deviation or median [1st quartile; 3rd quartile]

*ACE* angiotensin-converting enzyme, *ARB* angiotensin receptor blockers, *CBF* cutaneous blood flow, *CKD* chronic kidney disease (EPIdemiology collaboration formula), control, sham session (diastole synchronized compression/decompression session at 5 mmHg), *DBP* diastolic blood pressure, *DSCD* diastole synchronized compression/decompression session at 65 mmHg, *HDL* high-density lipoprotein, *LDL* low-density lipoprotein, *NA* not applicable, *PU* perfusion units, *SBP* systolic blood pressure

^a^Assessed using SphygmoCor^®^

**Table 2:** Patient characteristics for study 2

	Total	DSCD/Control	Control/DSCD	p-value
	(n = 38)	Arm 1 (n = 17)	Arm 2 (n = 21)	
Sex (Male/Female)	21/17	8/9	13/8	0.360
Age (years)	62.4 ± 7.6	62.8 ± 8.1	62.2 ± 7.4	0.811
Weight (kg)	93.3 ± 18.0	93.0 ± 14.9	93.6 ± 20.6	0.918
Height (cm)	168.6 ± 10.3	167.8 ± 10.5	169.3 ± 10.3	0.669
Body mass index (kg/m^2^)	32.8 ± 5.7	33.1 ± 5.6	32.6 ± 6.0	0.765
Waist circumference (cm)	112.1 ± 12.4	113.2 ± 9.6	111.3 ± 14.5	0.632
Smokers	3 (7.9)	2 (11.8)	1 (4.8)	0.577
HbA1c (%)	7.24 ± 1.22	7.29 ± 1.43	7.20 ± 1.06	0.883
HbA1c (mmol/mol)	56.52 ± 14.53	56.16 ± 15.65	56.80 ± 13.94	0.883
Diabetes duration (years)	8.4 [5.9; 15.7]	5.7 [4.8; 10.7]	10.4 [5.9; 15.7]	0.116
Fasting plasma glucose (mmol/L)	8.44 ± 2.90	8.93 ± 3.67	8.04 ± 2.10	0.399
Total cholesterol (mmol/L)	4.42 ± 1.02	4.43 ± 1.17	4.41 ± 0.91	0.951
HDL-cholesterol (mmol/L)	1.22 ± 0.31	1.17 ± 0.38	1.27 ± 0.24	0.379
LDL-cholesterol (mmol/L)	2.45 ± 0.91	2.38 ± 1.03	2.51 ± 0.82	0.663
Triglycerides (mmol/L)	1.75 ± 0.99	1.94 ± 0.73	1.59 ± 1.16	0.031
Non-HDL cholesterol (mmol/L)	3.20 ± 1.03	3.26 ± 1.16	3.15 ± 0.93	0.741
Urine albumin (mg/L)	9.9 [6.8; 23.9]	10.6 [6.2; 20.3]	9.2 [6.9; 23.9]	0.837
Plasma creatinine (µmol/L)	74.1 ± 15.2	75.5 ± 15.8	73.0 ± 15.0	0.635
Glomerular filtration rate (CKD, mL/min/1.73m^2^)	85.9 ± 14.6	83.0 ± 17.4	88.3 ± 11.9	0.303
Aortic SBP (mmHg)^a^	127.1 ± 8.9	128.9 ± 9.7	125.6 ± 8.2	0.268
Aortic DBP (mmHg)^a^	80.7 ± 8.7	80.1 ± 11.6	81.2 ± 5.6	0.717
Aortic pulse pressure (mmHg)^a^	46.4 ± 9.0	48.9 ± 11.4	44.4 ± 6.2	0.160
Rest left CBF (palmar hand) (PU)	92 [44; 143]	122 [48; 200]	88 [38; 128]	0.270
Brachial artery diameter (mm)	3.94 ± 0.65	3.89 ± 0.76	3.99 ± 0.56	0.666
FMD (%)	3.78 ± 2.01	4.08 ± 1.95	3.50 ± 2.06	0.379
Insulin treatment	14 (36.8)	7 (41.2)	7 (33.3)	0.618
Statins	23 (60.5)	12 (70.6)	11 (52.4)	0.254
Fibrates	2 (5.3)	1 (5.9)	1 (4.8)	1.000
Antiplatelet treatments	15 (39.5)	6 (35.3)	9 (42.9)	0.635
ACE-inhibitor	12 (31.6)	6 (35.3)	6 (28.6)	0.658
ARB	15 (39.5)	7 (41.2)	8 (38.1)	0.847
Beta-blockers	11 (28.9)	3 (17.6)	8 (38.1)	0.282
Calcium-channel blockers	11 (28.9)	4 (23.5)	7 (33.3)	0.721
Other antihypertensive drugs	13 (34.2)	4 (23.5)	9 (42.9)	0.212

Data are presented as numbers, percentages (%), mean ± standard deviation, or median [1st quartile; 3rd quartile]

*ACE* angiotensin-converting enzyme, *ARB* angiotensin receptor blockers, *CBF* cutaneous blood flow, *CKD* chronic kidney disease (EPIdemiology collaboration formula), control no session was performed, *DBP* diastolic blood pressure, *DSCD* diastole synchronized compression/decompression session at 65 mmHg, *FMD* flow-mediated dilation, *HDL* high density lipoprotein, *LDL* low-density lipoprotein, *PU* perfusion units, *SBP* systolic blood pressure

^a^Assessed using SphygmoCor^®^

### Microvascular blood flow response to one DSCD session

**Fig. 2:**
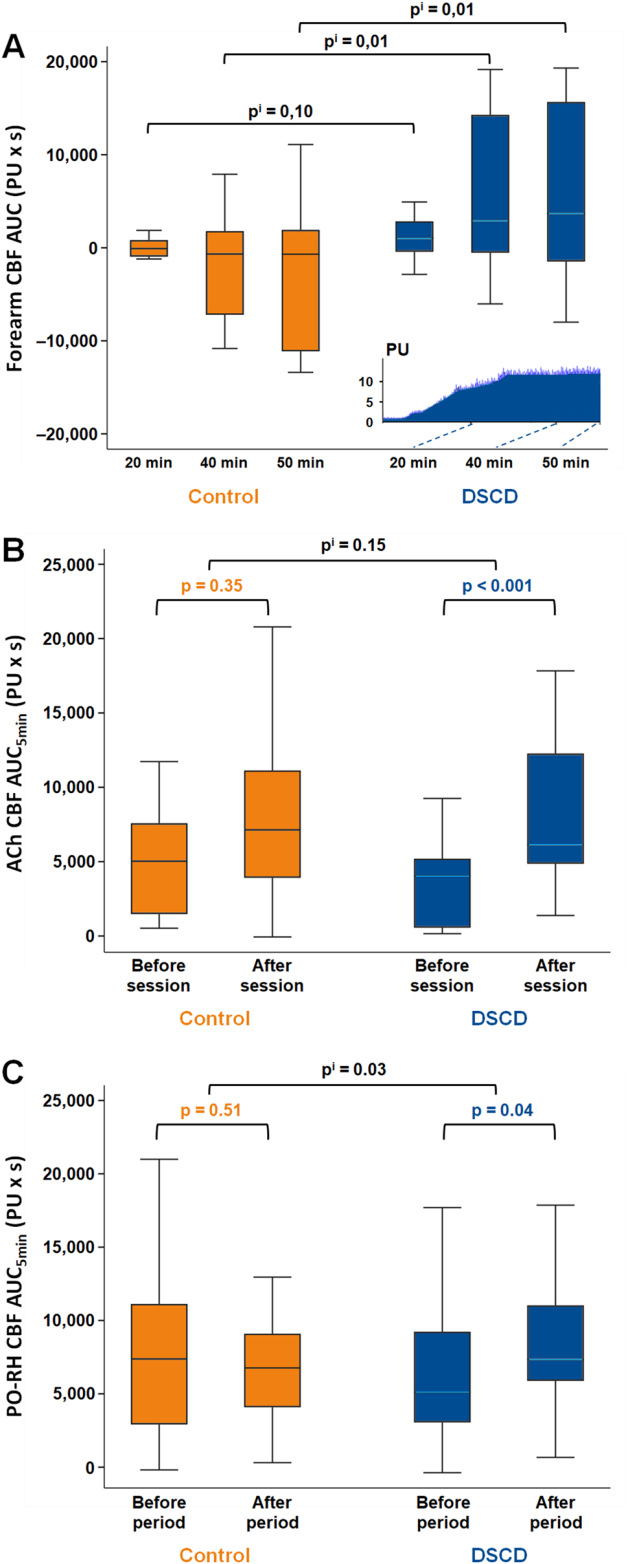
Effects of DSCD/control sessions on microcirculatory blood flow and endothelium function. **A**: Response of forearm CBF during and after one DSCD or Control session (Study 1). ACh; acetylcholine, AUC; area under the curve, CBF; cutaneous blood flow, control; simulated session, DSCD; diastole synchronized compression/decompression session at 65 mmHg, PU; perfusion units. AUC was calculated for 20 min during the session (AUC-20 min, from T60 to T80min) (p^i^ = 0.10), 40 min (20 min during and 20 min after session) (AUC-40 min, from T60 to T100min) (p^i^ = 0.01) and 50 min (20 min during plus 30 min after session) (AUC-50 min, from T60 to T110min) (p^i^ = 0.01). Note that the ACh test was performed 30 min before the DSCD/control sessions. Thus, baseline CBF was not restored when DSCD/control sessions started. During and after a session, CBF decreased in the control group due to the progressive return to the baseline CBF (with negative values for AUC-40 min and AUC-50 min), whereas CBF increased after DSCD session despite withdrawal effect of ACh (with positive values for AUC-40 min and AUC-50 min) highlighting the effect of DSCD session. p-value: comparison between groups (DSCD vs control) adjusted for period and sequence, p^i−2^: comparison between groups (DSCD vs Control) adjusted for period, sequence and initial BMI**.** In the cartouche: Illustration of CBF microcirculation increase in a 61 year-old woman, BMI of 27 kg/m^2^, on metformin and gliclazide treatment. When adjusted to the initial BMI, p^i−2^ was 0.1 for AUC-20 min, 0.06 for AUC-40 min and 0.08 for AUC-50 min. These results must be interpretated with caution in view of the number of patients and the conditions of application of the models implemented. **B**: Changes in ACh CBF AUC5min after one DSCD or Control session (Study 1). Forearm CBF was recorded for 5 min after ACh administration on the forearm 25 min before and after DSCD/control sessions. p-value: comparison between before and after session. p^i^: interaction between time (after session compared to before session) and group (DSCD compared to control) adjusted for period and sequence, p^i−2^: comparison between groups (DSCD vs Control) adjusted for period, sequence and initial BMI”. When adjusted to the initial BMI, p^i−2^ was 0.145 for AUC-20 min. **C**: Changes in PO-RH CBF after three months of DSCD sessions versus control (Study 2). PO-RH; post-occlusive reactive hyperaemia. PO-RH CBF was measured on left palmar hand. PO-RH CBF AUC5min was calculated from 5 min CBF measurement after a 5 min brachial artery occlusion. The test was performed before periods 1 and 2 and again after periods 1 and 2 (24 h after the last DSCD session for the DSCD period). p-value: comparison between before and after period. p^i^: interaction between time (after period compared to before period) and group (DSCD compared to control) adjusted for period and sequence; p^i−2^: interaction between time (after compared to before) and group (DSCD compared to Control) adjusted for period, sequence and initial body mass index; p^i−3^: interaction between time (after compared to before) and group (DSCD compared to Control) adjusted for period, sequence and change in total physical activity. When adjusted to the initial BMI, p^i−2^ was 0.032, and the change in total physical activity, p^i−3^ was 0.073

In Study 1, the mean DSCD CBF AUC-40 min and AUC-50 min were significantly higher compared to control sessions (p^i^ = 0.01) (Fig. 2A).

### Endothelium function changes in response to acute or chronic DSCD

In Study 1, ACh AUC5 min increased after the DSCD session (p < 0.001) but not after Control sessions (p = 0.35) (increase of the ACh AUC5 min of 192% [39; 720] in DSCD vs 145% [− 4.5; 298] in the control) (Fig. 2B), with a trend towards an interaction Group × Time (p^i^ = 0.15), while RHI remained unchanged (Table 3). In Study 2, PO-RH CBF AUC5 min increased after the DSCD period (p = 0.04) but not after Control period (p = 0.51) (increase of 43% [1; 155] vs − 3% [− 38; 22] change) with a significant interaction time × treatment (p^i^ = 0.032) (shown in Fig. 2C). FMD, brachial artery diameter and VTI did not differ after DSCD or after the Control period (recorded 24 h after the last DSCD session of the DSCD period) (Table 3).

**Table 3:** Effect of single and multiple treatments by DSCD on endothelium function (small or middle-sized arteries) (study 1 and study 2)

	Acute treatment (Study 1) (n = 16)								
	Control session			DSCD session			p^i^	p^i−2^	p^i−3^
	Before	After	p-value	Before	After	p-value			
Reactive hyperaemia index	1.90 ± 0.50	2.11 ± 0.40	0.176	2.07 ± 0.33	2.22 ± 0.48	0.298	0.839	0.874	NA

	Chronic treatment (study 2) (n = 38)								
	Control period			DSCD period			p^i^	p^i−2^	p^i−3^
	Before	After	p-value	Before	After	p-value			
Brachial artery diameter (mm)	3.91 ± 0.60	3.97 ± 0.65	0.547	4.00 ± 0.67	3.96 ± 0.57	0.527	0.332	0.332	0.165
FMD (%)	4.38 ± 2.09	4.17 ± 2.11	0.579	3.78 ± 1.67	3.76 ± 2.05	0.961	0.670	0.678	0.490
VTI (cm)	81.0 ± 21.0	84.3 ± 23.0	0.338	80.7 ± 22.0	84.8 ± 21.7	0.161	0.851	0.855	0.479
SSRH (dyn/cm^2^)	67.8 ± 20.6	67.9 ± 22.2	0.895	66.7 ± 19.5	70.4 ± 23.7	0.246	0.431	0.433	0.229

Data are presented as mean ± standard deviation. p-value: comparison between before and after session (Study 1) or period (Study 2); p^i^: interaction between time (after compared to before) and group (DSCD compared to Control) adjusted for period and sequence; p^i−2^: interaction between time (after compared to before) and group (DSCD compared to Control) adjusted for period, sequence and initial body mass index; p^i−3^: interaction between time (after compared to before) and group (DSCD compared to Control) adjusted for period, sequence and change in total physical activity. Control: simulated session (Study 1) and no session (Study 2)

*DSCD* diastole synchronized compression/decompression session at 65 mmHg, *FMD* brachial flow-mediated dilation, *NA* not applicable, *SSRH* shear stress in response to reactive hyperaemia, VTI speed of the hyperaemia response brachial artery diameter, FMD and VTI were recorded 24 h after the last DSCD session of DSCD period

### Biological parameters and body composition

**Fig. 3:**
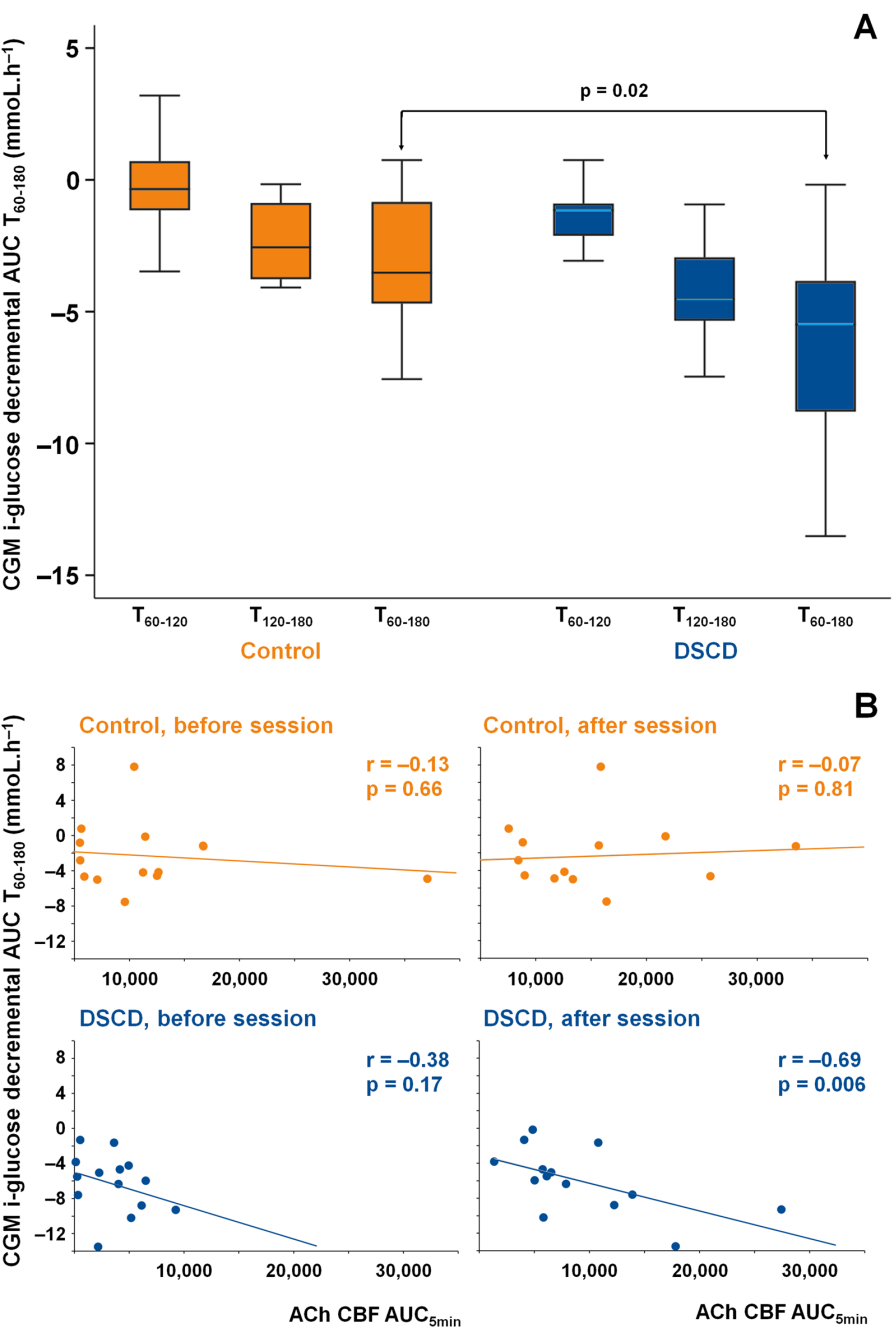
Effects of DSCD/control sessions on interstitial glucose and endothelium function. **A**: Decrease of interstitial glucose after one DSCD/control session (Study 1) for continuous glucose measures (CGM) i-glucose decremental AUCs. AUC, area under the curve; CGM, continuous glucose measures; Control, simulated session; DSCD, diastole synchronized compression/decompression session at 65 mmHg, i-glucose; interstitial glucose. Decremental AUC illustrates the decrease of i-glucose (CGM recording). Decremental AUCs were calculated for one hour after the start of DSCD/control session (AUC from T60 to T120minutes), for the next hour (T120 to 180 min) and for the 2 hours after the start of DSCD/Control session (T60 to 180 min). No crossover and period effects were observed allowing calculating the variance of the 2 h i-glucose decremental AUC between the control and DSCD (p = 0.02). **B**: Correlation between i-glucose decremental AUC and ACh CBF AUC5min. AUC; area under the curve, CGM; continuous glucose measures, control, simulated session, DSCD; diastole synchronized compression/decompression session at 65 mmHg; i-glucose, interstitial glucose. Breakfast was eaten 110 min before start of sessions (see Fig. 1). The Spearman’s correlation coefficient (denoted r) was calculated between i-glucose decremental AUC T60-180 and ACh CBF AUC5min after a single 20 min DSCD session. No significant correlation was found before the DSCD/control sessions

In Study 1, after the end of the sessions [(T80) (130 min after breakfast)], plasma glucose was lower after DSCD than after control sessions with a borderline significance (p^i^ = 0.054) (Table 4). The i-glucose decremental AUC for 60 and 120 min after start of session was greater after DSCD compared to the Control (for 120 min, p = 0.02) (Fig. 3A), still significant after being adjusted for the period, sequence, and ACh CBF AUC5min before or after a session (p < 0.001 for both). Additionally, i-glucose decremental AUC for 120 min correlated significantly with ACh AUC5min (r = − 0.69, p = 0.006) after DSCD sessions and not after control sessions nor before the sessions (Fig. 3B) and did not correlate with the changes in basal CBF.

**Table 4:** Changes in biological parameters and body composition (for study 1 and study 2)

Plasma and CGM interstitial glucose (i-glucose)				
Acute treatment (Study 1) (n=15)				
	Control session		DSCD session	p^i^
Fasting plasma glucose (mmol/L)^a^	8.78±1.94		8.04±2.38	0.532
Plasma glucose after session (mmol/L)^a^	13.58±2.09		11.01±2.54	0.054
i-glucose just before session (mmol/L)	11.45±3.20		12.06±2.09	0.528

Biological parameters and body composition (Impedance/PH metry)							
Chronic treatment (Study 2) (n=38)							
	Control period		p-value	DSCD period		p-value	p^i^
	Before	After		Before	After		
Fasting plasma glucose (mmol/L)	7.9 ± 2.0	8.1 ± 2.9	0.785	8.2 ± 3.2	8.5 ± 2.7	0.314	0.630
Insulin (pmol/L)	16.1	17.8	0.946	14.7	16.9	0.866	0.939
	[8.6; 29.6]	[10.3; 23.2]		[8.6; 25.8]	[10.0; 28.4]		
HOMA-IR index	5.0	5.7	0.533	5.4	6.1	0.059	0.512
	[2.8; 10.0]	[3.6; 11.1]		[2.7; 10.1]	[3.6; 12.2]		
TyG index^b^	9.16±0.54	9.24±0.67	0.383	9.29±0.78	9.30±0.60	0.902	0.673
HbA1c (%)	7.35±1.26	7.37±1.19	0.876	7.32±1.33	7.37±1.33	0.583	0.855
HbA1c (mmol/mol)	56.84 ±13.81	57.04 ±13.03	0.876	56.52 ±14.53	57.01 ±14.53	0.583	0.855
Total cholesterol (mmol/L)^b^	4.02±0.80	4.23±0.89	0.095	4.34±0.97	4.24±0.77	0.341	0.061
HDL-cholesterol (mmol/L)^b^	1.28±0.30	1.22±0.28	0.084	1.23±0.33	1.29±0.32	0.058	0.011
LDL-cholesterol (mmol/L)^b^	2.00±0.59	2.22±0.77	0.075	2.27±0.74	2.16±0.60	0.297	0.041
Triglycerides (mmol/L)^b^	1.70±0.87	1.98±1.32	0.069	2.23±2.08	1.88±0.94	0.226	0.073
Non-HDL cholesterol (mmol/L)^b^	2.74±0.72	3.01±0.93	0.038	3.11±1.00	2.95±0.74	0.151	0.013
Body fat mass (kg)	38.6±10.1	38.7±9.0	0.865	38.4±9.8	38.7±9.6	0.445	0.805
Lean mass (kg)	56.8±12.7	56.9±12.5	0.844	57.1±13.0	55.3±13.3	0.216	0.233
Total water (L)	46.7±6.9	46.7±6.1	0.957	47.0±6.9	46.8±6.6	0.591	0.749
Extra-cell water (L)	20.6±3.9	21.9±5.1	0.154	21.4±3.3	20.8±2.1	0.185	0.071
Intra-cell water (L)	27.1±4.5	25.9±5.3	0.166	26.7±4.2	27.0±3.5	0.345	0.091

Data are presented as mean ± standard deviation or median [1st quartile; 3rd quartile]. p-value: comparison between before and after period; p^i^: interaction between time (after period compared to before period) and group (DSCD compared to control group) adjusted for period and sequence, Control; simulated session (Study 1) and no session (Study 2)

*DSCD* diastole synchronized compression/decompression session at 65 mmHg, *HDL* high density lipoprotein, *HOMA-IR* Homeostatic model assessment of insulin resistance: Insulin (mU/L) x plasma glucose (mol/L)/22.5, LDL low-density lipoprotein, *TyG* index triglycerides-glucose index

^a^Fasting plasma glucose measured before session; Plasma glucose after session measured after the end of the 20 min session (130 min after breakfast)

^b^Biological criteria for two patients who started statin treatment during study were excluded from the analysis

In Study 2, the changes in lipid parameters differed after DSCD and control periods with an increase in HDL cholesterol (p^i^ = 0.011), a decrease in LDL-cholesterol (p^i^ = 0.041) and non-HDL cholesterol (p^i^ = 0.013), and a trend towards a decrease in triglycerides (p^i^ = 0.073) after DSCD session periods, while these parameters changed in an opposite way for the control group. These results remained unchanged when the initial BMI was adjusted (p^i2^ = 0.011, 0.041, 0.013 and 0.073 respectively) and were mostly unchanged when adjusted to the change in physical activity (p^i3^ = 0.023, 0.062, 0.020 and 0.078). There was no significant correlation between the changes in endothelial function and the changes in lipid parameters. There was no difference for the changes in glycemic parameters or for body fat and lean mass between the DSCD and control periods. Extra-cell water decreased and intra-cell water increased mildly after the DSCD period, while the opposite was seen after the control period (p^i^ = 0.071 and p^i^ = 0.091, respectively) (Table 4).

### Quality of Life

In Study 2, SF-36 Vitality and Physical component scores improved after the DSCD period compared to the control period (p^i^ = 0.030 and p^i^ = 0.080, respectively). No changes were recorded for the other SF-36 items. IPAQ total physical activity (moderate to vigorous activity) increased after DSCD period (p < 0.001) and did not change after the control period (p = 0.350) (Table 5), while time spent in sedentary behavior did not change in any period (Data not given).

**Table 5:** Changes in Quality of Life and physical activity (study 2)

	Chronic treatment (Study 2) (n=38)						
	Control period			DSCD period			p^i^
	Before	After	p-value	Before	After	p-value	
SF-36							
Physical component score	45.4±8.9	44.8±10.1	0.412	43.6±9.5	45.4±8.8	0.072	0.080
Mental component score	47.2±10.6	50.8±8.1	0.004	47.5±8.9	51.0±8.4	0.003	0.936
Physical functioning	74.5±24.2	75.9±24.8	0.188	70.5±26.1	75.1±24.5	0.093	0.295
Mental health	69.3±17.9	72.5±16.8	0.086	68.1±17.1	74.4±16.1	0.007	0.263
Vitality	57.9±19.2	60.1±18.8	0.249	54.2±19.5	62.6±16.1	<0.001	0.030
Social functioning	73.7±23.6	80.9±20.1	0.002	72.0±19.4	77.6±20.8	0.066	0.654
Role limitations due to physical problems	75.7±34.6	77.0±36.5	0.814	76.3±31.8	81.6±30.6	0.196	0.584
General health	59.7±21.0	59.5±22.1	0.927	54.9±21.6	58.6±18.4	0.127	0.199
Role limitations due to emotional problems	67.5±43.5	82.5±33.5	0.016	72.8±37.9	82.5±34.4	0.044	0.489
Bodily pain	58.1±28.0	61.4±26.1	0.137	55.2±27.0	65.0±25.8	<0.001	0.103
IPAQ							
Total physical activity (moderate to vigorous activity, METmin/week)	2586 [852; 6255]	3698 [1386; 7119]	0.350	2066 [1152; 3630]	3759 [1866; 6228]	<0.001	0.114

Data are presented as mean ± standard deviation or median [1st quartile; 3rd quartile]. p-value: comparison between before and after period, p^i^: interaction between time (after period compared to before period) and group (DSCD compared to Control) adjusted for period and sequence. Control no session, DSCD diastole synchronized compression/decompression session at 65 mmHg, IPAQ international physical activity questionnaire, MET equivalent of the metabolic task (equal to 3.5 ml of O_2_/kg/min with 1 MET equal to at rest O_2_ consumption), SF-36 36-item short form survey

### Safety

No adverse events were recorded in Study 1 or during the 1,482 DSCD sessions performed in Study 2 except for cases reporting calf cramps of moderate to severe intensity in four patients that did not require treatment nor needed the DSCD sessions ceased (2.2% of the overall sessions involved).

## Discussion

To our knowledge these are the first controlled studies investigating both single and multiple DSCD sessions applied to the lower part of the body to assess its effects on microcirculatory blood flow, endothelium function and metabolic parameters of patients with type 2 diabetes. While Study 1 and Study 2 patients were not strictly similar in age and BMI, our findings show an improvement of cutaneous blood flow and endothelium function and post-prandial glucose levels after one 20 min session as well as an improvement of endothelium function, blood lipid profile, extra-cell hydration, SF-36 Physical and Vitality components and IPAQ total physical activity after three months of use.

In Study 1, baseline CBF levels (at rest and after acetylcholine) measured on the forearm were similar to published results in patients with poorly controlled type 2 diabetes [27]. Baseline CBF levels were higher in Study 2 which could be due to the characteristics of arteriovenous anastomoses at palmar sites due to it glabrous properties [28]. The microcirculatory blood flow increases after a single 20 min DSCD session (Fig. 2A: CBF AUC-50 min 3690 [− 667; 11,476] increase vs − 693 [− 7852; 1702] decrease after the control session) is consistent with a study reported in healthy volunteers under the same conditions (97 ± 106% increase of CBF peak, p < 0.01) [16]. Regarding endothelium function, the mean RHI level at baseline in Study 1 (2.0 ± 0.3) was close to that which we previously reported in healthy subjects (2.3 ± 0.5) [29]. This may account for the lack of improvement after one DSCD session as compared to Control session in patients with type 2 diabetes. However, the trend to an improvement of skin CBF response to ACh in Study 1 is consistent with the improvement of PO-RH after 3 months of DSCD treatment in Study 2 despite the different sites investigated for CBF responses, in other words, non-glabrous skin (Study 1) and glabrous skin (Study 2) which was shown to be more responsive to stimuli [30, 31]. FMD, which explores endothelium-dependent NO-mediated dilatation of large arteries, showed enormous inter-subject variability and low reproducibility [32, 33]. In Study 2, mean FMD at baseline (3.78 ± 2.01) was closer than previously observed in healthy or overweight subjects (3.95 [1.43; 5.25] and 4.25 [1.74; 5.56], respectively) and far better than previously reported in patients with type 2 diabetes [34]. This, together with a managed glycemic control and other risk factors might explain why repeated DSCD sessions did not change the endothelium function of large arteries.

The improvement of microcirculation flow (Study 1) and endothelium function (both in Study 1 and 2) was recorded at a site away from the DSCD suit (on the forearm or the palmar hand vs the lower part of the body) and lasted at least 30 min after one DSCD session or 24 h after the last session of a 3 month DSCD period and strongly denotes systemic effects. These effects were recorded independently of blood pressure and heart rate changes, vago-sympathetic activity, cardiac output and peripheral vascular resistance (measured in Study 1, data not shown). This supports the mechanical effect of DSCD on endothelial cells, by evoking an EC shear stress effect with diffusion of vasoactive substances such as NO in blood flow. However, a DSCD effect on the smooth muscle cells cannot be excluded since a sodium nitroprusside test was not performed. The improvement of endothelium function was associated with a decrease in extra-cellular hydration after DSCD versus an increase in the control in Study 2 consistent with the limb volume decrease recorded after DSCD sessions in patients with lymphedema [35].

In both studies, significant metabolic changes occurred. In Study 1, after a single DSCD session, plasma glucose measured 130 min after a standard breakfast was significantly lower and the decrease of i-glucose was greater than the same measurements taken after one control session. In Study 2, there were no changes in glycemic parameters (fasting glucose and HbA1c) or insulin resistance index (HOMA-IR), however, post-prandial glucose was not measured. These results are in line with a study showing a glycemic control improvement after external counter-pulsation sessions at 300 mmHg in patients with type 2 diabetes. However, that study did not include a strict control group treated by simulated intervention(s) [13].

In type 2 diabetes, there is strong evidence for impairment of endothelial function [36, 37]. Associations between insulin resistance/endothelial dysfunction and insulin sensitivity/basal endothelial NO production have been reported in patients with metabolic and cardiovascular disorders [38]. This was corroborated by the effect of peroxisome-proliferator–activated receptor-γ agonists on NO production in type 2 diabetes patients [39]. Therapeutic interventions in animal and human studies betoken complex and reciprocal relationships between endothelial function and insulin sensitivity [40]. Likewise, a systemic NO bioavailability blockade in healthy subjects was shown to significantly impair glucose tolerance by increasing plasma insulin clearance and depressing insulin secretion [41]. This supports the idea that glucose homeostasis is linked to vascular health.

With regard to the relationship between microcirculation and glucose metabolism, NO bioavailability increases muscle microcirculation leading to increase in glucose consumption and decrease in plasma glucose [42]. In Study 1, this event was less noticeable due to the fact that our patients were tested under resting conditions, which substantially limits muscle glucose needs as well as the temperature-based high activity of type-4-glucose-transporter (GLUT4) and glucose consumption [43].

A correlation was found between the magnitude of i-glucose decremental AUC after a standard breakfast and endothelium function after the DSCD session in Study 1. However, this was not seen with microcirculatory flow increase. This finding suggests that EC stimulation during DSCD triggered an i-glucose decrease which was more likely to be a result of the effect of shear stress on EC glycolysis as proposed by in vitro studies [44]. Thus, DSCD sessions which increase shear stress and impact EC may improve endothelial function in patients with type 2 diabetes and subsequently improve post-prandial glucose levels. This is of great interest since it was recently reported that the post-prandial microvascular blood flow response of muscles is poor in normo-glycemic patients with a positive family history of type 2 diabetes and in those who suffer from type 2 diabetes compared to healthy subjects [45]. This alteration in post-prandial microvascular response is probably related to microvascular insulin resistance which likely plays an important role in glucose disposal by controlling the rate of glucose and insulin delivery to myocytes and is involved in type 2 diabetes pathogenesis. Our study may suggest a role of skin microcirculation in post-prandial glucose disposal. However, it is more probable that the improvement in skin microcirculation might be the sign witnessing a concomitant improvement in muscle microcirculation, which is the major mechanism for glucose uptake in post-prandial periods. Further studies are needed to demonstrate increased muscle microcirculation and glucose consumption after DSCD sessions.

The increases in endothelium function in Study 2 was associated with concomitant improvement of lipid profiles including a non-HDL cholesterol decrease and a trend towards lower triglycerides. While some observational studies suggest the role of lipid disorders, particularly high triglycerides, non-HDL cholesterol and low HDL-cholesterol in diabetic micro-angiopathic complications [46, 47], the role of shear stress or NO on lipids remains unknown. The changes in lipid parameters observed after the DSCD might result from the increase in vitality and physical activity observed in Study 2 patients, or from a yet unexplored effect on lipids stemming from the stimulation of endothelial function as indicated by the role of endothelium and NO in protecting vessels from atherosclerosis based on in vitro studies [48]. It may be important to note that these results were obtained in physically-active patients who spent little time in sedentary behavior (median of 2500 MET. minutes per week) in agreement with current guidelines. When adjusted to the change in physical activity, the improvement in lipid parameters remained mostly unchanged which stands in favor of the role of DSCD-induced endothelium function improvement on lipid changes. Despite the protective effects of such a lifestyle against cardio-metabolic complications, DSCD sessions were effective at improving endothelium function and lipid profiles, while the absence of improvement in HbA1c might be the result of relatively good glycemic control at study inclusion. Lastly, DSCD sessions were well tolerated without any adverse events reported. The safety and acceptability of DSCD (no patient withdrawal in our studies) are also supported in current physiotherapy practices (Data collected on 1515 patients with more than 7850 DSCD sessions, Stendo Company Internal Safety Regulatory Report).

Strengths of these studies primarily focused on the methodology used. Both Study 1 and Study 2 cohorts were randomized, with two cross-over arms (Arm-1 and Arm-2) and included a simulated intervention (DSCD session at 5 mmHg) in Study 1, at a variance with trials performed with EECP. A large range of cardiovascular indicators were explored and concomitant treatments remained unchanged in almost all of our patients. Moreover, in Study 1, assessments were performed in a post-prandial state after a standardized breakfast.

Our study had limitations. In Study 2, patients showed a relatively good metabolic profile which might have precluded further HbA1c improvement. In addition, glucose was not measured post-prandial which limited conclusions about the metabolic effects of DSCD. Another limitation of this study was that the different methods/stimuli/sites of measurements were applied for assessing the vascular function in Study 1 and Study 2, limiting us to compare the acute and chronic impact of DSCD. External assessment should be performed in order to confirm the acute and long-term benefits of DSCD with a larger sample size, and to evaluate whether these effects persist for a few days or weeks after the sessions. The time to maximum brachial artery dilation in Study 2 was also not analyzed and could be a parameter to measure in future studies. In addition, the effects in patients treated by the new glucose-lowering drugs must also be assessed in the future.

## Conclusion

Our study showed that in patients with type 2 diabetes, single and multiple DSCD sessions were well accepted and tolerated. The improvement of endothelium function with this non-invasive treatment may be critical due to the key role that endothelial dysfunction plays in the pathogenesis of type 2 diabetes and its complications. These findings provide the basis for quantifying a potential benefit of DSCD on long-term glucose and lipid metabolism and its preventive effect on microvascular complications and offer a strong rationale and promising perspective for conducting clinical trials to test the effects of DSCD on diabetic complications including foot ulcers.

## Data Availability

The data sets generated and/or analyzed during the current studies are available from the corresponding authors by reasonable request and in compliance with EU Regulations for the protection of personal data.
